# Exploring the Epistemological Significance of Qualitative Research in Behavior Analysis

**DOI:** 10.1007/s40617-025-01127-z

**Published:** 2026-01-05

**Authors:** Angela Arnold-Saritepe, Erin Leif, Amarie Carnett, Sarah Leadley, Rebecca Sharp, Russ Fox

**Affiliations:** 1 School of Psychology, University of Auckland, Auckland, New Zealand; 2 School of Educational Psychology and Counselling, Monash University, Melbourne, Australia; 3 School of Psychology, University of Waikato, Hamilton, New Zealand

**Keywords:** Qualitative inquiry, Behavior analysis, Social validity, Cultural inclusivity, Thematic analysis

## Abstract

In the evolving field of applied behavior analysis, there is a growing recognition of the value that qualitative research brings to understanding and improving interventions (Burney et al., *Perspectives on Behavior Science*, 46, 185–200, 2023; Leif et al., *Journal of Applied Behavior Analysis, 57*(3), 542–559, 2024). Our group of academics and practicing behavior analysts aim to share our journey towards incorporating qualitative approaches in our research and practice, underscoring their importance and application in enhancing our scientific endeavors. This article will guide readers through the process of qualitative inquiry, from thinking of words as data through to an introduction to qualitative inquiry methods and how to collect and analyze data. We then discuss some of the ethical considerations around qualitative research (researcher reflexivity, centering participant voice and cultural responsivity) and conclude with highlighting the value of mixed methods research, as we work towards enhancing the social validity of behavior-analytic interventions. Through sharing this journey, we are hoping our behavior-analytic peers will recognize that the path to including qualitative approaches in their research is not daunting and will lead to valuable insights into how we meet the needs of those we serve.

Applied behavior analysis research and practice has traditionally been dominated by quantitative methods, emphasizing observable and measurable behaviors. However, there has been a recent emergence of qualitative studies authored by behavior analysts, reflecting a burgeoning interest in the subjective experiences of individuals affected by behavioral interventions (Anderson et al., 2022; Burney et al., 2024a, 2024b; Leif et al., 2023). Our collective experiences of both acceptance and rejection from our peers within the applied behavior-analytic community have prompted reflexive inquiries into the legitimacy and value of qualitative approaches in our field. We posit that the heart of applied behavior analysis research and practice is inherently linked to qualitative research through the shared objective of understanding and improving human behavior (Wolf, 1978).

Qualitative research is an inquiry process of understanding a social or human process (Creswell & Poth, 2025). For behavior analysts, comprehending the lived experiences of those we support is paramount—not only in relation to the interventions we implement but also regarding the broader behavioral changes we seek to facilitate. This article advocates for integrating qualitative research to support a more nuanced understanding of behavior and its contextual influences, ultimately enriching the quality and efficacy of our scientific practices.

## Words as Data

Qualitative researchers analyze textual data, often in the form of verbal behavior recorded as permanent product (e.g., interviews and self-reports), to answer research questions of interest (Burney et al., 2024a, 2024b). Although behavior analysts may already collect such verbal behavioral data as part of their practice through indirect assessments (e.g., closed-ended and open-ended interviews), these words are typically used to more accurately identify the functionally relevant variables in the environment that are controlling behaviors of interest (Cooper et al., 2020). Furthermore, these data are often then combined with direct observations of the environments of interest to support an experimental examination of the variables identified (Saini et al., 2020). A hesitancy to extend the use of words as data exists in the behavior-analytic community. This may be due in part to a lack of reliability of words as data when compared to direct observational data (Baer et al., 1987). Furthermore, behavior analysts have been trained in and work within a scientific community that has created strong contingencies that maintain a cultural practice of avoiding inferring causal explanations for behavior based on the content—or topography—of verbal behavior of individuals, instead viewing such verbal behavior as instances of behavior in and of themselves. Even as the philosophical, conceptual, and technological developments related to verbal behavior within behavior analysis have continued (e.g., derived relational responding, acceptance and commitment therapy), researchers and practitioners have emphasized the importance of understanding verbal behavior in context and cautioned against inferring any described functional relations based on the topography of verbal statements describing behavior and the environmental variables alone (see Sandoz et al., 2021, for a more detailed description). It is important to note that words as data still hold great promise within behavior-analytic research and practice, with Burney et al., (2024a, 2024b) making the case that qualitative research still aligns with the core dimensions of behavior analysis. In particular, they noted that qualitative research focuses on socially significant phenomena and explores meaningful change, while describing in clear procedural detail what was done, when, and by whom, as well as proceeding with the important goals of generalizing findings cautiously. Thus, qualitative research is congruent with the applied, technological, and generalizable dimensions of applied behavior analysis as outlined by Baer et al., (1968).

Qualitative research is focused on different goals than quantitative research, and as such, different approaches are needed (Burney et al., 2023, 2024a, 2024b). As noted earlier, the need for a deeper comprehension of the lived experiences of those we support is a priority for many applied behavior analysts and practitioners. To truly comprehend such phenomena, we must, as behavior analysts, engage with the words or verbal behavior that individuals themselves use to describe who they are, what they care about, their experiences of direct contingencies, and their understandings of the world around them. Fortunately, there are historical examples of behavior analysts engaging with words as data to understand such phenomena. For example, engaging in Goldiamond’s nonlinear contingency analysis involves—among other steps—asking the individual being supported, “Where do you want to go?” (Layng et al., 2021). In doing so, the practitioner collects words as data that reflect self-discriminated and reported valued outcomes that can be incorporated alongside analyses of the environmental contingencies and used to support shared plan creation, shared progress monitoring, and shared evaluation of a plan.

Furthermore, as noted by Burney et al. (2023), the growing inclusion and understanding of derived relational responding in behavior analysis and the conceptual implications of these repertoires of responding (e.g., generative language learning, transformation of stimulus function, and rule-governance) may also increase the value of words as data. In addition, words as data may also provide an indication of how an individual self-discriminates (understands) and reports private events that form valued verbal behaviors such as their identity or sense-of-self (see McHugh et al., 2019, for a detailed description). As noted above, caution in using self-reported verbal behavior as descriptions of functional relations in the real-world is advised. Nevertheless, understanding that the individual’s learning history has shaped their self-report (Hineline, 2018; Sandoz et al., 2021) may allow for qualitative research to explore variables of interest as it relates to values, identity, and experience in a manner consistent with the broader field.

## Methodological Approaches in Qualitative Research

Behavior analysts venturing into qualitative research often employ open-ended survey questions and interviews, methods commonly used to explore questions about lived experiences via qualitative designs including narrative, grounded theory, ethnography, phenomenology, participatory, and generic approaches (see Leif et al., 2023). Every approach to qualitative inquiry offers unique strengths and is suited to different research questions and epistemological stances (Creswell & Poth, 2025). How does a researcher, particularly one new to qualitative inquiry, decide which of these approaches to use? The answer to this lies primarily in the research focus. What follows is a brief overview of six qualitative approaches including a brief discussion on generic qualitative research. We have deliberately left the discussion on generic qualitative research (also referred to as basic qualitative research) for last because generic qualitative research either combines several known methodologies or forges its own path from the research question, aligning with no particular methodology (Caelli et al., 2003).

### Narrative Research

Narrative inquiry originates from a plethora of fields (literature, history, anthropology, sociology, education, and psychology) that have all developed their own approaches to narrative inquiry (Kim, 2016). The essence of the narrative research approach is for the researcher to explore the lived experiences of an individual or group of individuals through the collection and analysis of stories to communicate information or make a point. It is based on the premise that humans make sense of their lives through narrative, and that these narratives are shaped by, and embedded within, personal, social, and cultural contexts. In terms of methodology, narrative inquiry typically begins with the co-construction of stories between the participant and the researcher (Creswell & Poth, 2025). This collaborative process involves open-ended conversations, interviews, and/or observations in which participants share meaningful experiences from their lives. Researchers must engage deeply and respectfully with the context of these experiences—including physical, social, emotional, temporal, and cultural dimensions—because these influence not only what is remembered and shared, but also how experiences are interpreted and given meaning (Clandinin, 2023). Once data are collected, participants’ narratives are “restoried” by the researcher. This involves organizing and synthesizing the shared experiences into a coherent narrative framework that reflects the essence of the participant’s story. The researcher then analyzes these narratives by identifying themes, patterns, or categories across or within stories. There are many challenges to this because the researcher must be aware of how their own positioning may affect restorying. Positioning, or positionality, refers to the effect of the researcher’s identity on the research. Positionality is identified by being aware of the researcher's position in relation to the topic under investigation, the research design, the context and the process (Savin-Baden & Major, 2013). The power imbalance between the researcher and participant is also inherent, as are ethical issues around story ownership (Pinnegar et al., 2006).

Burney et al., (2024a, 2024b) used narrative inquiry to understand how parents experience engagement in child-focused behavior-analytic interventions. Fifteen parents who had experienced child-focused interventions were interviewed and four themes were developed: (1) the system as the environment; (2) what parents bring; (3) what clinicians bring; and (4) where engagement happens. The study suggested that for interventions to be effective applied behavior analysts should attend to antecedent conditions for parent engagement rather than focusing on treatment adherence.

### Grounded Theory Research

Narrative inquiry focuses on individual stories where the objective of grounded theory research is not just to describe but to generate a theory for a process or an action. The researcher generates an explanation or theory of a process, and data is generated from people who have experienced the process (Glaser & Strauss, 2017). Grounded theory research originated in sociology in the 1960s when Glaser and Strauss posited that theories should be grounded in data from the field (Glaser & Strauss, 2017). Grounded theory research focuses on a process or action that occurs over time (for example, to determine what factors affect mental health diagnoses for people with intellectual disabilities). The researcher then seeks to develop a theory of this process by interviewing those with an understanding of the process. A theoretical model of the process is developed through induction. Grounded theory is useful when a theory is not available to explain a process and when a theory may be needed to explain how people are experiencing a phenomenon. The reader is directed to Corbin and Strauss (2014) for a framework in conducting grounded theory research.

The usefulness of grounded theory to the applied behavior analyst can be seen in a recent study that looked at the lived experiences of adolescents and young adults that engaged in muscle-building behaviors (Ganson et al., 2023). The aim of the study was to understand the process of initiation and continuation of muscle building behaviors using a grounded theory methodology. Semi structured interviews were conducted with 33 participants, data underwent initial and focused coding, and a grounded theory arose from the data and the researchers' interaction with the data. A process theory of engagement in muscle-building that moved through historical context and behavior initiation through to moving in and out of preservation and intensification phases of muscle building behaviors. The theory was underpinned by the central theme of balancing aesthetic, health, and functional goals related to muscle-building behaviors. This study exemplifies how grounded theory can be used in behavior analysis to uncover complex processes and generate actionable insights where no adequate explanatory model currently exists.

### Ethnography

Ethnographic research seeks to provide in-depth cultural insights (Agar, 1996). As such, the participant pool is much larger than that seen in narrative or grounded theory inquiry, where data collection typically ends after data saturation (i.e., the researcher is hearing no more new information). An ethnography looks at an entire group that shares a culture, which may on occasion be small but it is often large. The ethnographer becomes immersed in the group, conducting observations and interviews while sharing the day-to-day life of the individuals involved in the group (e.g., de Carvalho et al., 2017; Hammersley & Atkinson, 2019). The ethnographer seeks to understand the behaviors, values, language, and beliefs of the group under study. There is no one way to conduct ethnographic research, but there are features common throughout ethnographic research (Creswell & Poth, 2025).

In ethnographic research the researcher focuses on developing a complete description of a cultural group. de Carvalho et al. (2017) adopted an ethnographic approach, based on a behavior-analytic framework, to explore variables controlling tagging behavior (a specific type of graffiti) in Brazil. First, the researcher joined participants who engaged in tagging across all of their daily activities, followed by conducting interviews. Four different cultural practices in tagging were identified and analyzed to identify reinforcing contingencies that could lead to an increase in prosocial behaviors and decrease in tagging.

### Phenomenological Research

Phenomenological research aims at gaining a deeper understanding of the experiences of life. It seeks to describe the experiences or common meaning of several individuals in relation to a concept or phenomenon (e.g., grief, raising a child with a disability, experiencing a traumatic brain injury). The researcher collects data from people who have experienced the phenomenon and develops a description of the experience. Phenomenology draws heavily on the writing of Edmund Husserl (Kockelmans & Husserl, 1994) and has a strong philosophical component to it. Phenomenological research methods are used across the health sciences. In more recent research, phenomenology has contributed to theoretical debates in disciplines such as disability studies and critical race theory (Zahavi, 2025). Although there are variations within phenomenology (e.g., hermeneutical [interpretive] and transcendental [descriptive]), most phenomenological studies place emphasis on the phenomenon to be studied, and the participants are individuals who have experienced the phenomenon (Creswell & Poth, 2025).

An interpretive phenomenological analysis (a qualitative approach that examines how people comprehend major life experiences; see Smith et al., 2022) was used by Burney et al. (2025) to explore the lived experiences of two behavioral clinicians who were also parents of children who had received behavioral interventions. The study suggests that clinicians who are humble, listen actively, and value lived experience will have greater success in meaningfully involving parents in interventions for their children.

### Participatory Action Research

Participatory action research (PAR) is often mixed methods (containing both qualitative and quantitative inquiry), but we have elected to include it here because we believe this is an important tool that behavior analysts can utilize to ensure we are indeed applied in our approach. PAR seeks to improve and understand human experience (see Kemmis & McTaggart, 1988). It differs from other approaches because it aims to improve well-being and reduce inequities by involving the people whose experience this is. In turn, these people can take action to improve their well-being (Baum et al., 2006). PAR also differs from conventional research in that the purpose is to enable action. This is achieved through a reflective cycle where participants collect and analyze data and then determine what actions should follow. A blurring of the line between researcher and the researched is an essential component of participatory action and the equal sharing of power is advocated. PAR is seen as useful in Indigenous health research because it has the potential to reduce the colonizing effect of conventional research on Indigenous people.

### Generic Qualitive Research

Generic qualitative research is a flexible approach to qualitative inquiry that focuses on exploring individuals' subjective opinions, attitudes, beliefs, or reflections on their experiences in the external world (Percy et al., 2015). Unlike the established qualitative traditions (e.g., narrative inquiry, grounded theory, ethnography, phenomenology, PAR), generic qualitative inquiry does not adhere to a specific methodology or philosophical framework, making it adaptable to various interpretive lenses such as pragmatism or postpositivism (Kennedy, 2016).

As generic qualitative approaches follow no fixed methodology, there have been criticisms for the potential of poorly thought-out frameworks, lack of rigor, and method slurring (Kahlke, 2014). These criticisms have led to the proposal that frameworks such as Persson’s (2006) VSAIEEDC model of analysis are appropriate for use when conducting generic qualitative research. The VSAIEEDC process is a seven-step cognition-based model of analysis allowing for reflexivity and rigor. Despite criticisms there are benefits to generic qualitative research, particularly when new questions arise that do not fit the established methodologies.

Generic qualitative studies can be found throughout the disciplines and in applied fields of practice (Merriam & Tisdell, 2015). The primary data collection methods are interviews, questionnaires, and observations. The questions asked are those that are relevant to the disciplines and data analysis involves the identification of recurring patterns, that characterize the data. The subsequent interpretation of the data will be the researchers understanding of participants understanding of the question of interest (see Merriam & Tisdell, 2015).

An example of the use of generic qualitative methods in applied behavior analysis can be found in Max and Lambright’s (2022) investigation of the fidelity of applied behavior-analytic interventions in schools. A questionnaire with open-ended questions was distributed to board-certified behavior analysts (BCBAs) working in schools and thematic analysis was used to analyze the data. The findings indicated that BCBAs did not have sufficient time for training and there was a lack of support from school administrators thus leading to lack of fidelity of program implementation and decreased outcomes for students. Use of generic qualitative approaches have also begun to be embraced by behavior analysts seeking to answer questions relating to social validity in intervention development (e.g., Guinness et al., 2024) and interventions (Anderson et al. 2022).

To summarize, we have briefly described six approaches of qualitative inquiry. How does a behavior analyst who wishes to step into qualitative research know which path to take? That depends on your question. We encourage you to read widely on methods of qualitative inquiry. You will see that there is no one correct method of research embedded in any form of qualitative inquiry. Table 1 provides a brief overview of some of the things you may wish to consider when embarking on qualitative inquiry.

**Table 1:** Considerations for the behavior analyst when embarking on qualitative research

Qualitative Research Method	Examples of Data Collection Methods	Considerations for Use	Example Research Questions
Narrative Inquiry (including storytelling, yarning)	• Open ended interviews • Participant observations • Documents • Biographies • Pictures • Digital storytelling or audio recordings • Journals or personal diaries • Focus groups (to capture shared narratives) • Artifacts or cultural objects related to the story	• Useful for capturing personal histories and lived experiences • Requires trust and rapport between researcher and participant • Often used in Indigenous and culturally responsive research	• How do Indigenous families experience behavior-analytic services in early childhood? • What role does personal storytelling play in shaping self-advocacy among autistic adults receiving behavioral supports?
Grounded Theory	• Interviews with people to achieve detail in the theory • Focus groups (to explore emerging concepts collaboratively) • Field notes from observations • Memos written during data analysis • Document analysis (e.g., policies, reports relevant to the process studied)	• Aims to develop theories based on participant experiences • Requires systematic coding and constant comparison of data • It can be useful for identifying patterns in behavior-analytic service delivery	• How do practitioners conceptualize ethical challenges in behavior-analytic practice? • What factors influence the long-term sustainability of school-wide PBIS implementation?
Ethnography	• Participant observations • Interviews • Documents • A single culture sharing group • Field notes with detailed contextual descriptions • Informal conversations (unstructured) • Video or audio recordings of natural interactions • Cultural artifacts, rituals, or symbols	• Requires immersion in the participant’s environment to understand behaviors in context • Time-intensive and demands cultural competence • Ethical considerations include informed consent and minimizing researcher bias	• How do behavior support practitioners integrate cultural considerations into intervention planning? • What are the daily challenges and supports experienced by staff in disability support services?
Phenomenology	• Life story interviews • Interviews with a range of people In-depth, semi-structured interviews • Reflective journals or diaries from participants • Focus groups (to explore shared lived experiences) • Written personal narratives	• Used to describe a phenomenon that has / is being experienced by a group of people • Emphasis on the phenomenon • Seeks to understand the essence of a lived experience	• The experience of adults with intellectual disabilities moving from the family home into supported living environments • Living with a child with extreme behavioral distress
Participatory Action Research (PAR)	• Workshops and collaborative meetings • Community forums or discussion groups • Photovoice or participatory photographs • Collaborative journaling or reflective logs	• Participants actively collaborate in research design and decision making • Promotes empowerment and ensures findings are relevant to the community • Ethical considerations include maintaining equitable power dynamics	• How can behavior-analytic services be co-designed to better support the self-determination of neurodivergent individuals? • What changes do community stakeholders recommend to improve the accessibility of behavior support services?
Generic Qualitative Research	• Semi- or fully structured interviews • Questionnaires • Written or oral questionnaires • Observations	• Define your approach clearly (generic does not mean vague) • Ensure methodological rigor • Be reflexive in your interpretation	• Learner’s perspectives on being placed in specialist education classes • Description of autistic adults’ experiences after EIBI

## Selecting a Qualitative Approach

As described above, qualitative research offers behavior analysts a varied set of methodologies to explore complex human experiences, organizational contexts, and social processes that underpin behavior and intervention outcomes. Each qualitative approach—narrative inquiry, grounded theory, ethnography, phenomenology, PAR, and generic—holds distinct epistemological foundations, aims, and methods, providing unique opportunities and challenges depending on the research question and context (Creswell & Poth, 2025).

Selecting an appropriate qualitative approach and method fundamentally depends on the research aims, the nature of the phenomenon under study, and the desired outcomes. When the primary goal is to center individual or community voices and cultural narratives, particularly to illuminate identity and lived experience, narrative inquiry provides a respectful and context-sensitive approach. For studies aiming to develop new theories or explanatory models about processes or behaviors where existing frameworks are insufficient, grounded theory offers a robust method to inductively build understanding. Ethnography is well-suited for research focused on understanding broader cultural and organizational contexts, especially through immersion and observation, delivering comprehensive insights into group behaviors and shared practices. When the research emphasis is on exploring the shared essence of experiential phenomena, such as the emotional or psychological dimensions associated with interventions, phenomenology facilitates a deep examination of meaning from the participants’ perspectives. Participatory action research stands out when collaborative knowledge generation and empowerment of participants are prioritized, especially within marginalized or underserved communities, by engaging stakeholders as co-researchers to drive meaningful change. Generic qualitative research allows behavior analysts to answer the questions that fall between traditional methodologies broadening our scope to self-reflect as a scientific discipline. Behavior analysts are encouraged to align their methodological choices carefully with their research questions, participant needs, ethical considerations, and intended impacts. Integrating these qualitative approaches with behavior-analytic principles can significantly enhance the field’s ability to generate meaningful, culturally responsive, and action-oriented knowledge that advances both scientific understanding and applied practice.

## Collecting Qualitative Data

Qualitative research employs a variety of methods to gather rich, detailed data. These include focus groups, observational studies, open-ended questionnaires, and structured or semi-structured interviews. For behavior analysts unfamiliar with these methods, a primer on their application and utility is essential (see Guest et al., 2013). Focus groups facilitate the collection of different perspectives through group discussions, whereas observational studies enable researchers to systematically observe and record behaviors in naturalistic settings. Open-ended questionnaires and interviews allow for the collection of detailed, narrative data from participants, offering a window into their subjective experiences. Personal journals, case notes, documents, and family/cultural artifacts also provide an opportunity for data collection.

## Strategies for Data Analysis

Once all your data have been collected, your research question and the data you have collected will guide your analysis. Common forms of qualitative analysis include, though are not limited to, content, narrative, discourse, and thematic analysis. Content analysis may include thematic analysis and looks at the patterns that emerge by grouping words, content, and themes (Krippendorff, 2019), whereas narrative analysis focuses on thematizing or composing the story (Josselson & Hammack, 2021). Discourse analysis investigates how language shapes meaning, social structures, and power dynamics. Thematic analysis seeks to identify the meaning behind the words. This is achieved by looking at repeating themes in the text (Braun & Clarke, 2023; Wolgemuth et al., 2025).

Table 2 provides an overview of the steps involved in conducting one type of qualitative analysis, *inductive thematic analysi*s. Inductive thematic analysis is a widely used approach for analyzing qualitative data, particularly when exploring lived experiences, social validity, and the contextual factors influencing behavior. This method, as outlined by Braun and Clarke (2006), involves systematically identifying patterns within qualitative data through an iterative process of coding and theme development. Unlike deductive approaches that test preexisting theories, inductive thematic analysis allows the researcher to actively and inductively generate themes from the data itself, making it particularly useful for exploratory research in behavior analysis. We have chosen to focus this section on inductive thematic analysis. If readers are interested in extending this approach to reflexive thematic analysis, which involves further reflexive and reoccurring engagement with the dataset please see Braun and Clarke (2019).

**Table 2:** Steps for conducting inductive thematic analysis (adapted from Braun & Clarke, 2006)

Step	Description	Rationale
Step 1: Become familiar with the data	Read and reread transcripts from interviews, focus groups, observational field notes, or case study data. Take initial notes on participants' perspectives, experiences, and contextual factors	Deep familiarity with the data helps capture the lived experiences of individuals engaging with behavior-analytic interventions, ensuring that their voices and perspectives are accurately represented
Step 2: Generate the initial codes	Systematically label and categorize key elements of the data, such as participant-reported barriers, facilitators, perceived effectiveness, and contextual influences on behavior	Organizing data into meaningful units allows for the identification of codes related to, for example, the acceptability, feasibility, and perceived impact, benefits, or adverse outcomes of interventions
Step 3: Organize codes into themes	Identify patterns in the data that capture significant aspects of participants’ experiences, such as common perceptions of intervention effectiveness, ethical concerns, or autonomy in decision-making	Themes help make sense of the data by grouping related codes into broader concepts
Step 4: Review the themes	Review the themes to ensure they accurately represent participant experiences, checking for consistency, overlap, or missing perspectives. Refine or adjust themes as needed	Ensures that the analysis authentically reflects the data (for example, that the perspectives of the direct consumers of behavior-analytic interventions are accurately captured), strengthening the relevance and applicability of findings
Step 5: Define the themes	Finalize and articulate each theme’s meaning, ensuring clarity in how they relate to the research question. Thematic maps (a map focusing on themes and their relationship) may be used	Provides a clear conceptual structure for analysis and prepares the themes for reporting
Step 6: Write up	Integrate themes into a coherent narrative, supported by data extracts, and discuss their significance in relation to the research question	The final step ensures that findings are clearly communicated, making them accessible to the intended audience

Coding is often the initial step in organizing data by grouping similar observations, phrases, or behaviors into categories. In qualitative research, a code is a label assigned to an aggregation of data into a smaller quantity of information (Creswell and Poth (2025). For example, in a qualitative study exploring the challenges practitioners face when attempting to reduce the use of restrictive practices in response to challenging behavior, codes included “safety concerns,” “fear of reducing restrictive practices,” “feeling on the part of caregivers that the restrictive practice was necessary,” and “reluctance to implement alternatives to restrictive practices” (Leif et al., 2023). A theme, on the other hand, is a broader pattern that the researcher creates from multiple codes and captures a significant concept within the data. Themes are created through an abstract interpretation of the more detailed codes, that look beyond the recurring elements to embody patterns that link the data to the research questions (Naeem et al., 2023). For instance, in the study described above, the codes were organized into the broader theme of “reluctance.”

Inductive thematic analysis aligns well with behavior-analytic research by offering insights into how interventions are perceived, implemented, and experienced in real-world settings. This method complements traditional behavior-analytic approaches, such as single-case research designs, in several ways. First, behavior analysts emphasize the importance of social validity—whether interventions are acceptable, feasible, and meaningful to individuals (Wolf, 1978). Thematic analysis provides a way to systematically capture participant perspectives, ensuring that behavior-analytic interventions align with client values and needs. Second, thematic analysis can be used alongside quantitative research methods to provide a more comprehensive understanding of behavior change. For example, a study measuring skill acquisition can be enriched with qualitative interviews exploring caregivers’ experiences implementing the intervention. Finally, many behavior-analytic interventions require collaboration with parents, educators, and practitioners from other disciplines. Thematic analysis helps identify barriers and facilitators to implementation, such as staff training needs, environmental constraints, or ethical considerations in restrictive practice use. By integrating inductive thematic analysis with behavior-analytic methodologies, researchers can gain a richer understanding of both the effectiveness and lived experiences of behavior interventions, ultimately contributing to more ethical, effective, and person-centered support.

## Ethical Considerations in Conducting Qualitative Research

### Researcher Reflexivity

Behavior-analytic researchers engaging in qualitative inquiry are encouraged to practice reflexivity—critically examining their own cultural positioning, biases, and potential influences on the research process. We understand this may be foreign to the behavior analysts who have immersed themselves in quantitative data. Reflexivity is a fundamental component of ethical and rigorous qualitative research, because it helps researchers recognize how their identities, experiences, and preconceptions shape the way they collect, analyze, and interpret data (Berger, 2015; Braun & Clarke, 2019; Finlay, 2002). This self-examination is particularly crucial when working with people from historically marginalized communities, because it ensures that research findings genuinely reflect the lived experiences of participants rather than being unintentionally filtered through the researcher’s own worldview.

Reflexivity is an ongoing, dynamic process rather than a one-time activity. It requires researchers to critically question their assumptions at every stage of the research process, from formulating research questions to conducting interviews, analyzing data, and presenting findings. Behavior analysts engaging in qualitative research can adopt several strategies to cultivate reflexivity:

1. Engaging in Reflexive Journaling—Researchers can maintain a reflexive journal throughout the research process, documenting their thoughts, emotions, assumptions, and any shifts in perspective. This practice allows them to track how their positionality evolves and helps ensure that interpretations remain participant-centered rather than researcher-imposed.
2. Seeking Consultation and Peer Debriefing—Regular engagement with cultural advisors, community members, and interdisciplinary colleagues can support researchers in critically examining their assumptions, surfacing overlooked perspectives, and deepening their interpretations of qualitative data (Cornish et al., 2023). These conversations are especially valuable for recognizing how language, power, and positionality influence the research process. Peer debriefing promotes ethical decision making and accountability by providing a structured space to question normative frameworks and ensure the research remains responsive and inclusive across contexts—not only in relation to specific cultural or historically marginalized groups, but in any setting where hierarchies or systemic inequities may shape participant experiences.
3. Integrating Participant Feedback—Involving participants in reviewing findings (e.g., through member checking; McKim, 2023) can enhance the credibility of qualitative research and ensures that interpretations accurately capture their perspectives. Researchers can present preliminary analyses to participants and invite them to provide clarifications or corrections, thereby fostering a more collaborative approach to knowledge production.
4. Critically Reflecting on Power Dynamics—Reflexivity requires an awareness of power imbalances between researchers and participants, particularly when working with marginalized communities. Researchers should critically examine how their role, background, and institutional affiliations may influence participant responses and take steps to minimize these effects (e.g., by adopting participatory research methods or involving community co-researchers).
5. Providing Transparent Methodological Descriptions—Clear, detailed descriptions of qualitative research methods in behavior-analytic literature enhance the practical application of findings and allow others to assess the study’s rigor. Explicit discussions of reflexivity within published research can also model best practices for future researchers (Braun & Clarke, 2006).

For behavior analysts conducting qualitative research, reflexivity is essential for ensuring that research is not only methodologically sound but also culturally responsive.

### Ensuring Cultural Responsiveness

Behavior-analytic interventions must be culturally responsive to be both effective and ethical. Cultural responsiveness involves recognizing, respecting, and integrating the cultural contexts, values, and lived experiences of the individuals we serve (Jimenez-Gomez and Beaulieu, 2022). Failure to consider cultural responsiveness can result in interventions that are misaligned with the needs of diverse communities, reducing their acceptability, uptake, and overall impact.

For Indigenous communities, qualitative methodologies provide a platform to honor Indigenous voices, challenge colonial research paradigms, and reduce power imbalances (Drawson et al., 2017). These approaches emphasize relationality, reciprocity, and community engagement, ensuring that interventions are not only evidence-based but also culturally meaningful and community-driven. Engaging Indigenous scholars and community leaders in research teams is essential for ensuring that research methods align with cultural values and protocols (Clark et al., 2023; Taylor et al., 2024; Walker et al., 2021). Indigenous qualitative research prioritizes Indigenous epistemologies, acknowledging that knowledge is relational, experiential, and embedded within cultural practices.

For example, within New Zealand Māori communities, *tikanga* (customary practices) shapes how research is conducted (Jones et al., 2006). Culturally respectful research practices include:

- *Mihimihi* (Introductions): Establishing relational connections before formal discussions.
- *Karakia* (Prayer): Beginning and ending research engagements with prayer to honor spiritual traditions.
- *Kai* (Sharing Food): Relationship-building through shared meals, fostering trust and reciprocity.

Researchers should approach interviews and discussions with cultural humility, recognizing the expertise of participants in their own lived experiences. This may involve adapting language, using culturally appropriate metaphors, and allowing space for storytelling rather than rigid question-and-answer formats. Autoethnography and storytelling are powerful qualitative methods that allow for a deeper exploration of personal and collective experiences. Yarning, a conversational method used in Indigenous research, facilitates storytelling in a way that respects cultural narrative traditions. Unlike structured interviews, yarning allows participants to weave in and out of stories naturally, enabling richer, more authentic insights into their experiences with behavior-analytic interventions. By listening to cues within yarning conversations, researchers can identify themes relevant to intervention development while honoring the storytelling traditions of participants. This method supports the creation of interventions that reflect community values and lived realities rather than imposing external frameworks that may be culturally misaligned. Using these methods allows researchers to capture deeper, more meaningful insights into participants’ experiences (Bessarab & Ng’Andu, 2010). Building genuine relationships with communities requires more than just obtaining informed consent; it involves reciprocity, shared decision making, and a commitment to using research for the benefit of the community (Vaughn & Jacquez, 2020).

Addressing power imbalances in research also involves ensuring that Indigenous researchers conduct interviews, because their presence and shared lived experiences can create safer spaces for participants to share their perspectives. A recent study exploring Chinese caregivers' attitudes to behavior-analytic therapy involved interviews conducted by a Chinese researcher, which allowed all interviews to be conducted in Mandarin (Pan et al., 2024). A further strength of this study included the author piloting the semi-structured interview initially with a Chinese caregiver of Autistic children and responding to feedback.

Data sovereignty is also critical for ensuring that research benefits communities and aligns with their values and priorities. In culturally responsive behavior-analytic research, this means involving communities from the outset in research design, including the formulation of research questions (Te Mana Rauranga Maaori Data Sovereignty Network, 2018). It also means ensuring that data are used for the explicit benefit of the community, with clear agreements on its applications. Finally, community members and research participants should be provided with access to their data, and researchers should hold collective discussions to co-analyze findings and determine next steps. This participatory approach empowers communities by ensuring that research is not extractive but instead contributes to positive, self-determined change.

Findings from Indigenous qualitative research have already informed significant changes in health care and behavioral support systems. For example, the voices of New Zealand Māori have influenced the development of more culturally attuned treatments for eating disorders (Clark et al., 2023) and pediatric feeding disorders (Taylor et al., 2024). In the context of applied behavior analysis, qualitative research can similarly inform the adaptation of interventions to enhance accessibility and relevance. It important to note that research should include individuals historically excluded from behavior-analytic services due to systemic barriers. By understanding the perspectives of those who have not yet accessed behavioral interventions, researchers can identify and address gaps in service provision, ultimately expanding access to culturally safe supports.

### Centering Participant Voices and Lived Experiences

An essential dimension of ethical qualitative research, particularly when conducted with individuals from historically marginalized communities, is the intentional and sustained centering of participants’ voices and lived experiences. In applied behavior analysis, this is especially critical in light of the documented concerns and critiques raised by autistic individuals and their allies about how their perspectives have historically been excluded or unreported or underreported in behavior-analytic research and practice (Anderson, 2023; Leaf et al., 2022). Qualitative research provides a vital opportunity to shift this legacy by engaging directly with those most affected by behavior-analytic interventions, policies, and systems.

Centering voice is both a methodological and ethical commitment to honoring the dignity, autonomy, and lived realities of participants, particularly those from marginalized communities. However, voice is not something that researchers “give” to participants. Rather, ethical qualitative research recognizes that participants already have voice and expertise in their own lives. The role of the researcher is to create the conditions in which that voice is heard, respected, and meaningfully engaged with (Lebenhagen, 2020). As noted, this involves moving away from extractive models of data collection toward more relational and collaborative approaches that foreground participant agency and interpretation, “The participant knows best” (Guerin, 2018, p. 257).

## Complementing Single-Case Research Design with Qualitative Approaches

Single case research design (SCRD) has long been the methodological gold standard within behavior analysis, for both its experimental rigor and capacity to demonstrate functional relationships at the individual level (Kazdin, 2021). However, SCRD’s quantitative focus on observable behavior and experimental control may overlook the rich contextual and experiential factors that influence outcomes in applied behavior-analytic research and practice. Integrating qualitative approaches alongside SCRD offers a complementary avenue to deepen understanding by capturing subjective experiences, environmental contingencies, and social dynamics that shape behavior Leaf et al., 2016). By combining qualitative approaches with SCRD, behavior analysts can produce more nuanced, contextually grounded data that enrich interpretation, guide responsive intervention adaptations, and enhance the sustainability of behavior change (Johnson & Christensen, 2020). This mixed methods integration could bolster the scientific and applied contributions of behavior analysis, advancing a comprehensive approach that respects both empirical rigor and the complexity of human experience.

One example of this within behavior analysis is the pursuit of social validity. Social validity centers on the acceptability and perceived importance of interventions and outcomes as judged by practitioners and stakeholders, including clients and their families (Leif et al., 2024; Wolf, 1978). In research, social validity is often measured after the intervention phase (i.e., treatment) is completed using rating scales (Leif et al., 2024). Although rating scales may offer efficiency and provide some insight into perceptions of social validity, they may not be accessible to all clients and may also limit our analysis of the elements of social validity (i.e., perception of goals, procedures, and outcomes). Qualitative methods provide powerful tools for exploring the elements of social validity by capturing the nuanced perspectives and subjective experiences of service recipients and stakeholders. Through interviews, focus groups, and open-ended questionnaires, researchers can gain further insights into the values, beliefs, and attitudes that shape clients' and stakeholders' perceptions of intervention goals, components, and outcomes. By understanding these perspectives, behavior analysts can tailor interventions to be not only effective but also socially acceptable and meaningful to ensure that the intervention and services resonate with and meet the real-world needs of those involved (e.g., service recipients, practitioners, stakeholders, and society).

There are a range of tools that can be used to help understand the needs of clients and guide interventions. The following section provides examples from the literature and suggestions to help guide practitioners in making informed decisions about measurement strategies. For example, Vogelgesang et al. (2016) introduced a mixed methods approach consisting of a standardized social validity questionnaire (Intervention Rating Profile-15), semi-structured teacher interviews, and regular email journal entries from the teacher to gain insight into perceptions of social validity for an intervention to increase self-monitoring for students with ADHD. Likewise, used a mixed method approach to assess social validity across student, parent, and teacher perspectives for an intervention aimed at improving self-management during homework tasks. For their measurement, students, parents, and teachers were asked to complete a Likert-scale questionnaire about their experience during the intervention. This assessment was followed by interviews with the teachers and students to gain more insight into their perceptions and the effects of the intervention. Last, Lambert et al. (2022) analyzed additional qualitative information (open-ended questionnaire responses) to evaluate the social validity of a university practicum addressing the challenging behavior of individuals with disabilities. In addition to Likert-scale questions, the questionnaire included open ended questions with prompts such as "What advice would you give to a parent in the [program]?" and "Before initiating services, I wish we had discussed...." Codes were derived based on participant responses (i.e., inductive) and thematic analysis was used to identify meaning in the data. The inclusion of qualitative data allowed the authors to reflect further on the social validity of the intervention. For example, some qualitative responses highlighted dissatisfaction with the program (considering it another “empty therapy” after lack of progress), in contrast with corresponding quantitative data (90% reduction in challenging behavior).

A mixed-method approach was also incorporated to assess the social validity perceptions of practitioners working within residential schools serving students with intellectual and developmental disabilities. A questionnaire that included questions across multiple categories (e.g., resources, roles and responsibilities, sense of support, satisfaction) was evaluated using 4-point Likert scales and open-ended questions to gain additional participant information related to their experiences. Thematic analysis was used to summarize the data across categories and topics. Although the responses to the open-ended questions tended to align with the ratings on the questionnaire, the authors highlighted that the use of open-ended questions can enhance social validity assessment and provide an opportunity for comparison of response patterns within the data collected.

Researchers have also provided guidance on the development of social validity measures. For example, Anderson et al. (2022) developed social validity questionnaires from the results of semi-structured interviews with caregivers of children with tube dependency (aged 3–14 years) who had received in-home interventions. The questionnaires then focused on items of most importance to caregivers, including novel items such as the benefits of behavioral feeding treatment beyond eating, spiritual/cultural beliefs, and the importance of the provider “believing” treatment would work. Interviews were analyzed using thematic (qualitative) analysis and textual (quantitative) analysis and the resulting themes were used to generate items for two social validity questionnaires. This example highlights the value of using a mixed method approach that uses qualitative methods to ensure content validity for the development of a question-based social validity assessment. Expanding beyond education, public health research has also used qualitative methods to assess social validity in health interventions. For instance, co-design approaches have been increasingly utilized to ensure that interventions align with the lived experiences and preferences of communities (Palmer et al., 2021). Co-design emphasizes collaboration between researchers and participants at every stage of the intervention process, ensuring that social validity is embedded in the design and implementation phases rather than assessed retrospectively. Incorporating qualitative research methods into social validity assessments offers behavior analysts deeper insights into how interventions are perceived, how they affect clients and stakeholders, and how they can be improved to maximize cultural relevance and effectiveness.

## Conclusion

Behaviorism, as we know it, will eventually die—not because it is a failure, but because it is a success. As a critical philosophy of science, it will necessarily change as a science of behavior changes. . . . (Skinner, 1969, p. 267)

In conclusion, we would like to invite the behavior-analytic community to continue to embrace change. We acknowledge the substantial value that qualitative approaches bring to behavior analysis while also recognizing their limitations. Qualitative research approaches enable the inclusion of underrepresented voices, providing a more comprehensive understanding of their experiences. Moving forward, the field of behavior analysis stands to benefit from adopting more participatory and collaborative approaches, leveraging qualitative approaches to enrich our scientific practices and better meet the needs of the communities we serve.

By integrating qualitative research approaches, behavior analysts can enhance the social validity, inclusivity, and cultural responsiveness of their interventions, ultimately fostering a more humane and effective practice. This article provides a roadmap for behavior analysts interested in incorporating qualitative approaches into their work, offering a comprehensive overview of methodological approaches, tools, and practical considerations. Through this endeavor, we aim to contribute to the growth and evolution of behavior analysis as a dynamic and inclusive field of study.

## Data Availability

Not applicable.
